# Pompe disease in China: clinical and molecular characteristics

**DOI:** 10.3389/fcvm.2023.1261172

**Published:** 2023-12-14

**Authors:** Jing Li, Xiaohe Shi, Bo Wang, David H. Hsi, Xiaoli Zhu, Shengjun Ta, Jing Wang, Changhui Lei, Rui Hu, Junzhe Huang, Xueli Zhao, Liwen Liu

**Affiliations:** ^1^ Department of Ultrasound, Xijing Hospital, Xian, Shaanxi, China; ^2^ Heart & Vascular Institute, Stamford Hospital, CT and Columbia University College of Physicians & Surgeons, New York, NY, United States

**Keywords:** Pompe disease, infant-onset Pompe disease, China, hypertrophic cardiomyopathy, echocardiography

## Abstract

**Background:**

Pompe disease (PD) is a rare, progressive, and autosomal recessive lysosomal storage disorder caused by mutations in the acid *α*-glucosidase gene. The clinical course and molecular mechanism of this disease in China have not been well defined.

**Methods:**

In this single-center cohort study, we investigated a total of 15 Chinese patients with Pompe disease to better understand the clinical manifestations, echocardiographic imaging and genetic characteristics in this population.

**Results:**

The median age of 15 patients at symptom onset was 5.07 months (1–24 months). The median age at diagnosis was 19.53 months (range: 3 to 109 months, *n* = 15). Average diagnostic delay was 13.46 months. None of the patients had received enzyme replacement therapy (ERT). Fifteen patients died at a median age of 24.80 months due to cardiorespiratory failure (range 3–120 months). Myasthenia symptoms and severe hypertrophic cardiomyopathy were universally present (15/15 = 100%). Global longitudinal strain (GLS) by echocardiography was significantly lower in these patients. After adjusting for gender, body surface area (BSA), left ventricular ejection fraction (LVEF), E/e'ratio, maximum left ventricular wall thickness (MLVWT), left ventricular posterior wall (LVPW), left ventricular outflow tract (LVOT)gradient, GLS was independently correlated with survival time (hazard ratio (HR) = 0.702, 95% confidence Interval (CI): 0.532–0.925, *P* = 0.012). In our cohort, we identified 4 novel GAA mutation: c.2102T > C (p.L701P), c.2006C > T (p.P669l), c.766T > A (p.Y256N), c.2405G > T (p.G802V). 12 patients were compound heterozygotes, and 4 homozygotes.

**Conclusions:**

Our study provides a comprehensive examination of PD clinical course and mutations of the GAA gene for patients in China. We showed clinical utility of echocardiography in quantifying heart involvement in patients with suspected PD. GLS can provide prognostic information for mortality prediction. We reported four novel mutations in the GAA gene for the first time. Our findings may improve early recognition of PD characteristics in Chinese patients.

## 1. Introduction

Pompe disease (OMIM 232300), also known as glycogen storage disease type 2 (GSD2) is a rare, progressive, autosomal recessive disorder caused by a deficiency of the lysosomal enzyme acid alpha-glucosidase (GAA; OMIM 606800), leading to lysosomal glycogen accumulation in many tissues, particularly respiratory, skeletal and cardiac muscles (1–3). Patients' age at the onset of Pompe disease and the rate of deterioration can vary considerably. First symptoms can present in infants, children, and adults. In clinical practice, Pompe disease has been classified in two main subtypes: infant-onset Pompe disease (IOPD), which is rapidly progressive with a typical hypertrophic cardiomyopathy, and the late-onset Pompe disease (LOPD), characterized by a milder disease progression and free from cardiac involvement (4, 5). The “classic” form applies to those infants who die within the first year of life, while the term “atypical” defines patients with milder myopathy who typically have respiratory failure between 12 and 18 months of age but can live longer (6).

Given the low prevalence of Pompe disease, very few cases of Pompe disease have been reported in China (4). There are relatively rare data describing the distribution of GAA mutations, phenotypes, disease course, and treatments for this disorder among individuals from China. The GAA gene is located on chromosome 17q25.2–q25.3 and contains 20 exons (1).

As of May 2023, more than 900 different variants had been listed in the Pompe Disease Mutation Database (http://www.Pompecenter.nl/). These gene mutations exhibit race specificity. In Thai patients, Amarinthnukrowh et al. reported that mutation c.1935C > A accounted for 80% of the mutant alleles and is the most common mutation in the Thai population (7). The c.1726G > A causes low GAA activity in normal individuals and is relatively common in Asian populations (8). While in Caucasian and Belgian patients, the most common mutation is c.-32-13T > G (4).

However, despite the increasing awareness of Pompe disease, data on PD in patients from mainland China are still limited. The objective of the present study was to identify the clinical manifestations, echocardiographic imaging and genetic characteristics of classic and atypical PD.

## 2. Patients and methods

### 2.1. PD patients

Fifteen patients with the classic infantile form and the more slowly progressive non-classic form Pompe disease were analyzed from May 2015 to December 2020 at the hypertrophic cardiomyopathy (HCM) clinic in the Department of Ultrasound, Xijing Hospital (Xi'an, China). Confirmative diagnoses were conducted by GAA genotyping and/or by peripheral blood smear evaluation. Clinical data were retrospectively collected from medical records. All participants were provided written informed consent. All investigations were conducted in compliance with the principles of the Declaration of Helsinki and were approved by the ethics committee of Xijing Hospital, Fourth Military Medical University (Xi'an, China). We obtained signed informed consent from patients' parents.

### 2.2. Echocardiographic examination

A complete two-dimensional and Doppler echocardiographic exam was performed in each patient according to the criteria of the American Society of Echocardiography (9). Echocardiographic studies were performed with the EPIQ 7C Ultrasound System (Philips Medical Systems) with a S5-1 and X5-1 transducer (1.0 to 5.0 MHz). The acquired data were digitally stored and subsequently analyzed by two observers who were blinded for the clinical data. All echocardiographic measurements were averaged from three beats. Left ventricular ejection fraction (LVEF) was determined using biplane Simpson's method (10). Ventricular septal thickness and left ventricular outflow tract (LVOT) gradients were assessed (11, 12). Body surface area (BSA) was calculated using the Mosteller formula (13). Diastolic function was evaluated and analyzed as previously described including left ventricular inflow pattern, and Doppler tissue imaging (including the E/e′ ratio) (14). We obtained 3D-STE strain and dyssynchrony parameters by off-line analysis using a TomTec software (4D LV analysis V.3.0; TomTec Imaging Systems, Munich, Germany). The 3D strain measurements included global longitudinal strain (GLS). GLS was averaged over 16 segments. The observers were blinded to clinical information. All strain and dyssynchrony values are expressed as percentages (15).

### 2.3. Enzymatic activity measurement of GAA

Human GAA (Lysosomal alpha-glucosidase) ELISA Kit was used to detect the activity of GAA enzyme. A 3.2 mm (1/8 in) DBS is incubated in the buffer solution containing internal standard and substrate provided by the kit, at 37°C for 18.5 h. And then water and NeoLSD Extraction Solution were added for extraction. 50 µl supernatant was transferred to a new well and dried with nitrogen. The enzymatic activity could be measured according to the signal response of the target analyte relative to the internal standard.

### 2.4. Mutation analyses

A GAA mutation analysis was performed in 15 patients with infantile-onset Pompe disease. All exons and intron/exon boundaries of the GAA gene were amplified and assessed by polymerase chain reaction (PCR) and direct sequencing. The obtained sequences were compared with the reference sequences NM_000152.4 to identify pathogenic mutations. All nucleotide differences between the patients and reference sequence were compared to the Single Nucleotide Polymorphism Database (dbSNP) of the National Center of Biological Information (NCBI) (16). The pathogenic nature of novel missense mutations was verified by direct sequencing of 300 alleles of unaffected individuals. To predict the effect of newly identified missense mutations, we used the Mutation taster (http://www.mutationtaster.org/) (17) and Polyphen2 software programs (http://genetics.bwh.harvard.edu/pph2/index.shtml). Multiple protein sequence alignment of vertebrate species [:Homo sapiens (accession no. NP_001073272.1), Pan troglodytes (accession no. XP_001160653.1), Macaca mulatta (accession no. XP_001109980.1), Canis lupus familiaris (accession no. XP_005624079.1), Bos Taurus (accession no. NP_776338.1), Mus musculus (accession no. NP_032090.3) and Rattus (accession no. NP_954549.1), Danio rerio (accession no. XP_001921957.2)] were performed by using Clustal Omega software (https://www.ebi.ac.uk/Tools/msa/clustalo) (18).

### 2.5. Statistical analysis

Statistical analysis was performed with SPSS version 26 (IBM Corp.). Continuous variables were expressed as mean ± SD, and categorical variables as frequencies and percentages. The continuous data were compared using the Student's t tests and Wilcox on-rank sum tests when assumptions were not met. For the categorical variables, either Pearson's chi-squared or Fisher's exact tests were used. In order to identify risk factors for mortality, cox proportional hazards regression analyses were used for each of the variables. Two-sided *P* < 0.05 was considered to be statistically significant. For the entire cohort, Kaplan–Meier analysis was used to estimate overall survival. Overall survival was defined as the time from the date of initial diagnosis of PD to the date of death or last follow-up. Receiver operating characteristic curves were used to propose a GLS cutoff for the composite outcome.

## 3. Results

### 3.1. Demographic and baseline characteristics

Demographic and baseline data for these patients are summarized in Table 1. Of the 15 Chinese patients included, the median age of 15 patients at symptom onset was 5.07 months (1–24 months). The median age at diagnosis was 19.53 months (range: 3 to 109 months, *n* = 15). Mean diagnostic delay was 13.46 months. Five patients had family history (33.33%). None of them had received enzyme replacement therapy (ERT). During follow-up, fifteen patients died at a median age of 24.80 months due to cardiorespiratory failure (range: 3–120 months). The most common symptoms were cardiomegaly, airway infections, muscle weakness and heart failure were the most common.

**Table 1:** Baseline demographics for the 15 patients with PD.

Gender	
Male	8 (53.55)
Female	7 (46.67)
Age at symptom onset (months)	5.07 (6.08)
Year of genetic diagnosis (months)	19.53 (36.35)
Age at death(months)	24.80 (39.78)
Family history	
Yes	5 (33.33)
No	10 (66.67)
Symptoms	
Cardiomegaly	11 (73.33)
Airway infections	7 (46.67)
Muscle weakness	6 (40.00)
Heart failure	5 (33.33)
Shortness of breath	4 (26.67)
Feeding difficulties	3 (20.00)
Short stature	1 (6.67)
Language disorder	1 (6.67)
Strephenopodia	1 (6.67)
Mental retardation	1 (6.67)
Poor hearing	1(6.67)

Values are mean ± SD or *n* (%). PD, Pompe disease; SD, standard deviation.

### 3.2. Echocardiographic findings

**Figure 1:**
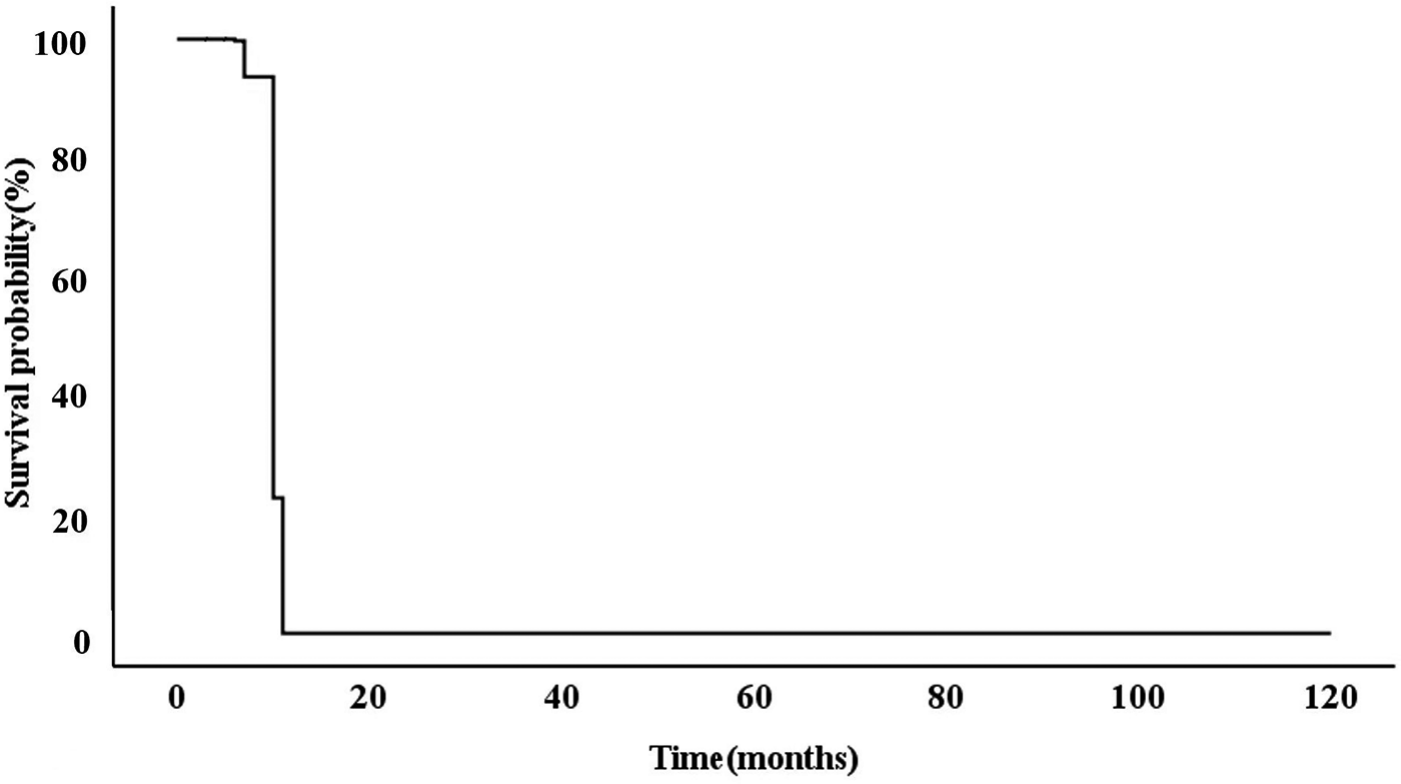
After adjusting for gender, BSA, LVEF, E/e’, MLVWT, LVPW, LVOT gradient, GLS was independently correlated with survival time (HR = 0.702, 95% CI: 0.532–0.925, *P* = 0.012). BSA,body surface area; LVEF, left ventricular ejection fraction; MLVWT, maximum left ventricular wall thickness; LVPW, left ventricular posterior wall; LVOT, left ventricular outflow tract; GLS, global longitudinal strain; HR, hazard ratio; CI, confidence interval.

**Figure 2:**
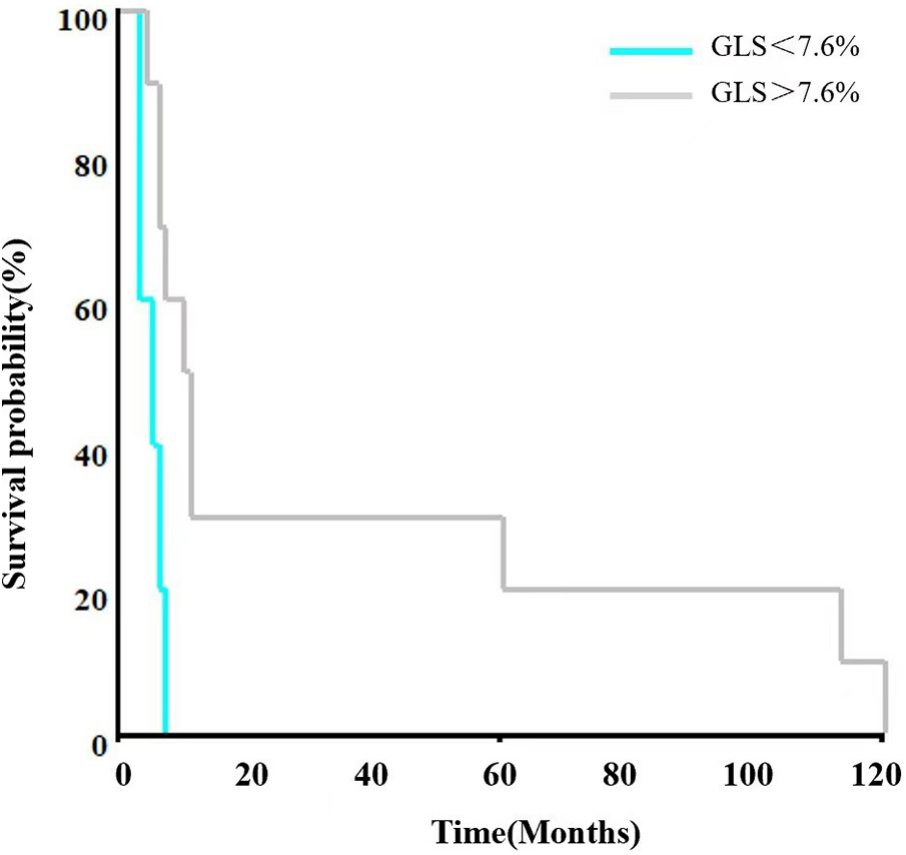
Kaplan–Meier representation of survival as stratified by GLS, patients with GLS baseline lower than 7.6% had a significant reduction in survival (HR = 3.266, 95%CI 0.861–12.391, *P* = 0.082). GLS, global longitudinal strain; HR, hazard ratio; CI, confidence interval.

All 15 patients had an HCM phenotype on presentation with a mean maximum left ventricular wall thickness (MLVWT) of 14.95 mm (range, 9.3–22 mm, Table 2). None of our patients had a left ventricular outflow tract gradient. Furthermore, LVEF was less than 50% in 7/15 (46.67%) of patients. Parameters from other echocardiogram available included mean (SD) left ventricular posterior wall (LVPW) 10.23(3.81) mm, mean (SD) LVOT gradient 3.27(1.49) mmHg, mean (SD) E/e’ ratio 24.23 (7.40) (Table 2). GLS was significantly lower in all 15 patients. After adjusting for gender, BSA, LVEF, E/e', MLVWT, LVPW, LVOT gradient, GLS was independently correlated with survival time (hazard ratio (HR) = 0.702, 95% confidence Interval (CI): 0.532–0.925, *P* = 0.012) (Figure 1). The calculated 7.6% cutoff value for GLS was determined by X-Tile software (19) (Supplementary Figure S1). The cumulative survival probability of patients with GLS < 7.6% was significantly lower than that of children with GLS > 7.6% (*P* >0.05) (Figure 2).

**Table 2:** Echocardiographic parameters for the 15 patients with PD.

ID	Gender	BSA (m^2^)	MLVWT (mm)	LVPW (mm)	LVOT gradient (mmHg)	SAM	LVEF (%)	E/e’ ratio	GLS
1	F	0.35	22	15	1	0	54	15.8	9
2	M	0.3	21	15	3	0	48	28.8	6.3
3	M	0.3	13	10	6	0	47	24.5	10
4	M	0.37	12	7	3	0	63	16.4	15.4
5	M	0.24	21	16	2	0	47	25.5	9.8
6	F	0.3	15	8	4	0	48	27.5	6.8
7	F	0.31	11	10	4	0	43	23.6	8.4
8	M	0.31	13	7	3	0	48	26.7	7.6
9	F	0.28	12	7	2	0	50	18.1	8.2
10	M	0.24	14	9	3	0	51	22.2	10.5
11	F	0.84	22	17	3	0	61	34.5	17.1
12	F	0.35	13	10	5	0	50	21.2	5.7
13	M	0.37	12	6	6	0	58	39.7	9.3
14	M	0.24	14	11	2	0	47	28.3	-
15	F	0.85	9.3	5.5	2	0	65	10.7	14.8
mean ± SD			14.95 ± 4.31	10.23 ± 3.81	3.27 ± 1.49	0	52.00 ± 6.67	24.23 ± 7.40	9.67 ± 3.50

PD, Pompe disease; ID, identity; F, female; M, male; BSA, body surface area; MLVWT, maximum left ventricular wall thickness; LVPW, left ventricular posterior wall; LVOT, left ventricular outflow tract; SAM, systolic anterior motion; LVEF, Left ventricular ejection fraction; GLS, global longitudinal strain.

### 3.3. Molecular characterization

**Figure 3:**
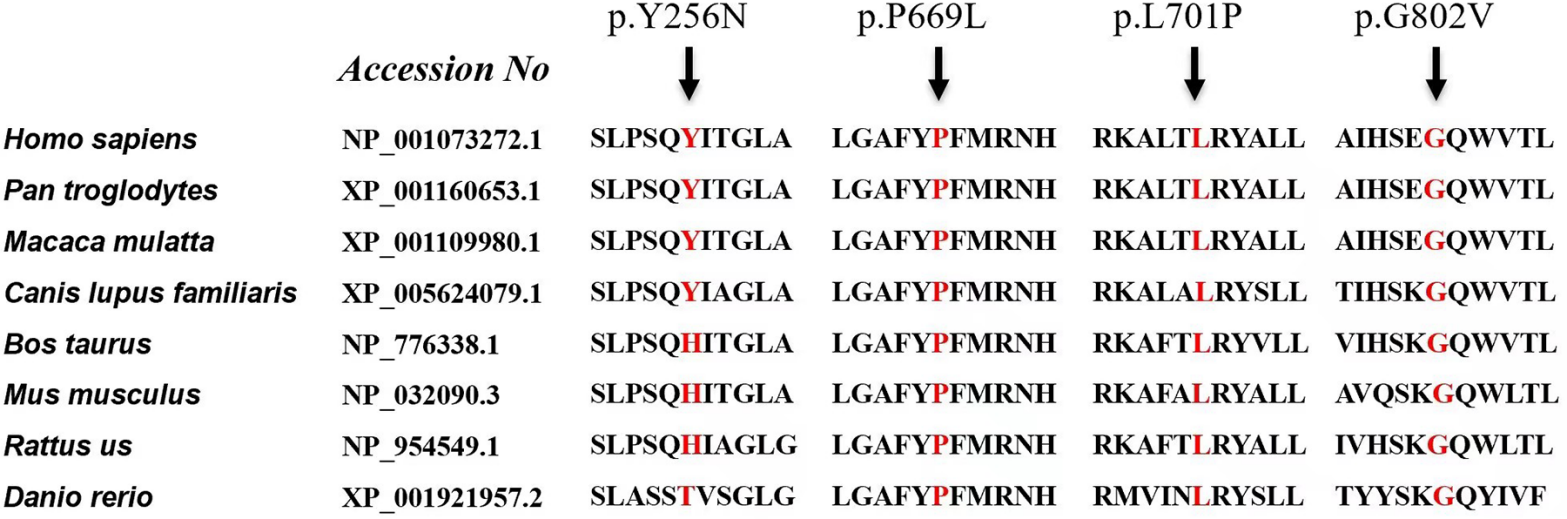
Conservative analysis. Protein sequence alignment of vertebrate GAA. GAA, acid alpha-glucosidase.

**Figure 4:**
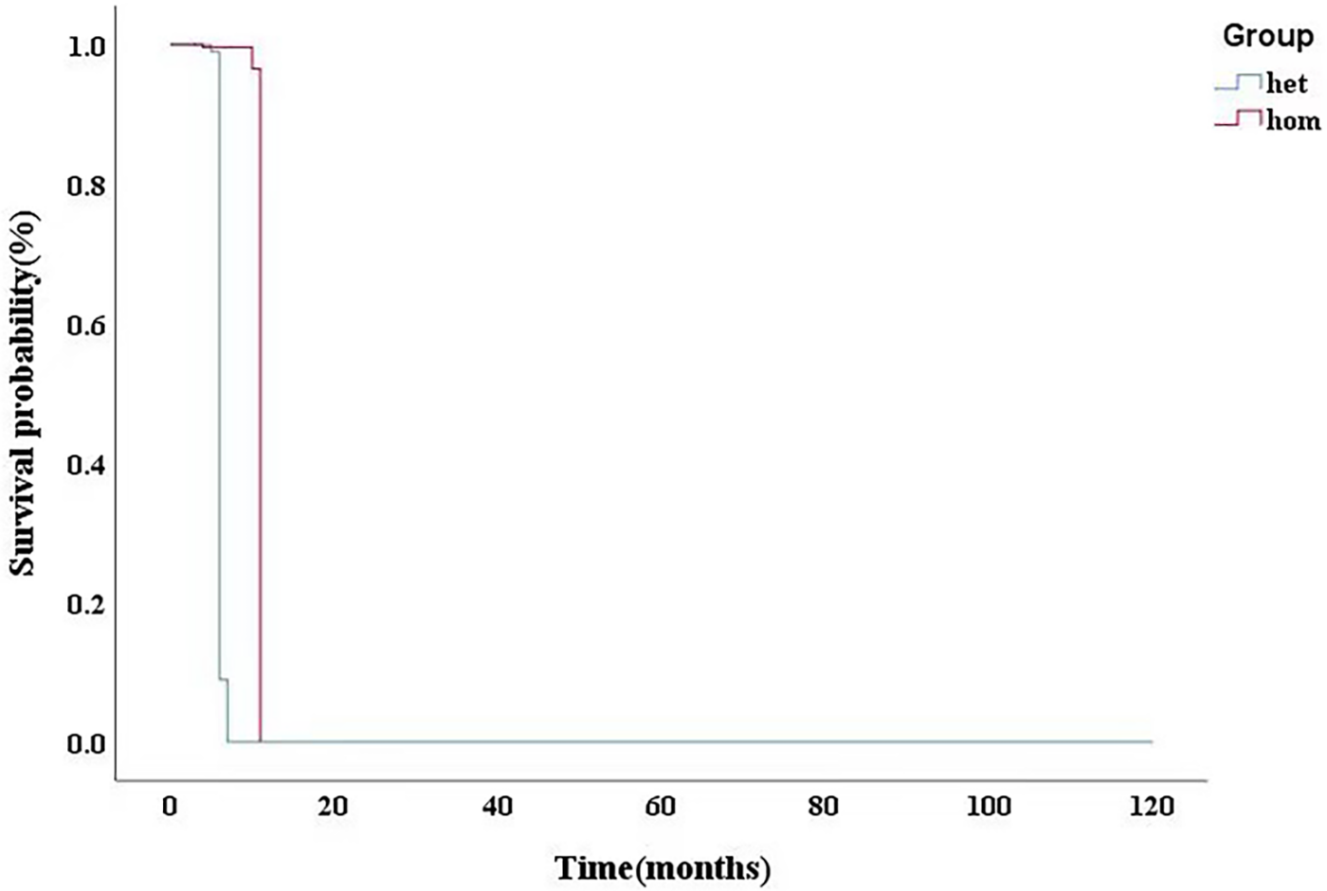
After adjusting for gender, BSA, LVEF, E/e’, MLVWT, LVPW, LVOT gradient, GLS, there was no significant difference in survival time between heterozygotes and homozygotes (*P* > 0.05). BSA,body surface area; LVEF, left ventricular ejection fraction; MLVWT, maximum left ventricular wall thickness; LVPW, left ventricular posterior wall; LVOT, left ventricular outflow tract; GLS, global longitudinal strain.

In our cohort, 12 patients were compound heterozygotes: 4 of them carried homozygous mutations in GAA. We identified 4 novel GAA mutation: c.2102T > C (p.L701P), c.2006C > T (p.P669l), c.766T > A (p.Y256N), and c.2405G > T (p.G802V) (Table 3). Comparative in silico analysis of the p.L701P, p. P669l, p. Y256N and p.G802V showed evolutionary conservation among vertebrate species of p.L701P, p. P669l and p.G802V residues. Based on the results of protein sequence alignments, the locations of mutation in p.Y256N are strongly conserved between the near species (Figure 3). After adjusting for gender, BSA, LVEF, E/e', MLVWT, LVPW, LVOT gradient, and GLS, there was no significant difference in survival time between heterozygotes and homozygotes (*P* > 0.05, Figure 4).

**Table 3:** Mutation spectrum of the GAA gene in 15 Chinese patients with PD.

ID	Exon number	Nucleotide change	Amino acid alteration	Mutation form	Origin	Pathogenicity
1	exon12	c.1669A > T	p.I557F	het	paternal	pathogenic
	exon14	c.1935C > A	p.D645E	het	maternal	pathogenic
2	exon12	c.1669A > T	p.I557F	het	paternal	pathogenic
	exon19	c.2662G > T	p.E888X	het	maternal	pathogenic
3	exon19	c.2725G > A	p.V909M	hom	parental	uncertain significance
4	exon7	c.1082C > T	p.P361l	het	maternal	pathogenic
	exon19	c.2662G > T	p.E888X	het	paternal	pathogenic
5	exon14	c.1933G > A	p.D645N	hom	parental	pathogenic
6	exon15	c.2102T > C	p.L701P	het	paternal	new
	exon19	c.2662G > T	p.E888X	het	maternal	pathogenic
7	exon14	c.2006C > T	p.P669l	hom	parental	new
8	exon14	c.1935C > A	p.D645E	hom	parental	pathogenic
9	exon9	c.1432G > A	p.G478R	het	maternal	pathogenic
	exon4	c.766T > A	p.Y256N	het	paternal	new
	exon16	c.2238G > C	p.W746C	het	maternal	pathogenic
10	exon9	c.1432G > A	p.G478R	het	paternal	pathogenic
	exon4	c.766T > A	p.Y256N	het	maternal	new
	exon16	c.2238G > C	p.W746C	het	maternal	pathogenic/likely pathogenic​
11	exon2	c.118C > T	p.R40X	het	maternal	pathogenic
	exon8	c.1280T > C	p.M427T	het	paternal	uncertain significance
12	exon13	c.1826dupA	p.Y6090_A610delinsX	het	-	pathogenic
	exon4	c.784G > A	p.E262K	het	-	pathogenic/likely pathogenic
13	exon12	c.1669A > T	p.I557F	het	-	pathogenic
	exon15	c.2237G > A	p.Trp746	het	-	pathogenic
14	exon17	c.2405G > T	p.G802V	het	maternal	new
	exon13	c.1844G > T	p.G615V	het	paternal	uncertain significance
15	exon4	c.796C > T	p.P266S	het	-	pathogenic
	exon7	c.1082C > T	p.P361l	het	-	pathogenic

PD, Pompe disease; GAA, acid alpha-glucosidase; ID, identity; het, heterozygote; hom, homozygote.

### 3.4. Echocardiogram and pathology

**Figure 5:**
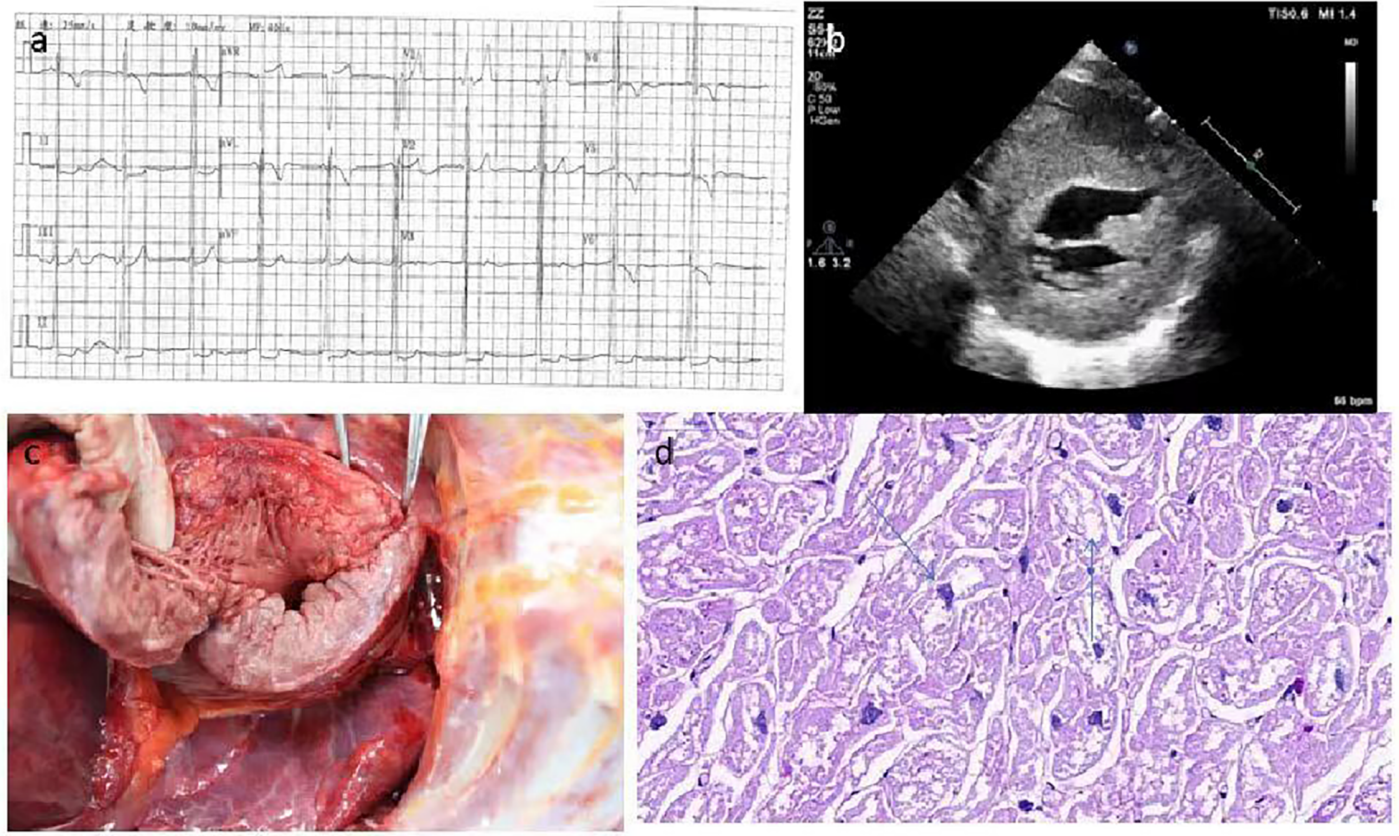
The gross cardiac specimen and PAS staining of cardiac ventricular septum of case 11. (**A**) Electrocardiogram examination suggested left ventricular hypertrophy. (**B**) Echocardiographic images suggested myocardial hypertrophy. (**C**) Severe myocardial and endocardial thickening, papillary muscle thickening. (**D**) A large number of glycogen particles in cells, Cytopalsmic vacuolar degeneration. PAS, Periodic Acid-Schiff.

Patient 11 was diagnosed at the age of 9 years with Pompe disease, and passed away at the age of 10 years. She had difficulty in unsteady wide-based gait and frequent falls at 12 months, and slimer than her peers (Supplementary Table S1). As she grew older, her family found that she had lameness, language problems, moderate mental retardation, poor hearing, and short stature. She had a comprehensive examination in Xijing Hospital when she was 9 years old. Biochemical examinations reported that creatine kinase (CK): 1085U/l (41–330 U/L), creatine kinase myocardial band (CKMB): 97 U/L (0–18 U/L), alanine transaminase (ALT): 117 U/l (10–35 U/L), aspartate transaminase (AST): 225 U/l (10–50 U/l) were elevated. Electrocardiogram examination suggested left ventricular hypertrophy (Figure 5A). GAA mutation analysis showed “c.118C > T” and “c.1280T > C” (Table 3). Two-dimensional echocardiographic images of patient 11 demonstrate severe left ventricular hypertrophy (Figure 5B). Tissue Doppler imaging illustrates severely reduced septal E′ (3.3 cm/s). Autopsy in patient 11showed glycogen accumulation in various parts of cardiac system. The gross cardiac specimen and Periodic Acid-Schiff (PAS) staining of cardiac ventricular septum showed that there were a large number of glycogen particles in cells, cytoplasmic vacuolar degeneration (Figures 5C,D).

### 3.5. Genetic analysis: prenatal diagnosis of GAA gene and follow-up

**Figure 6:**
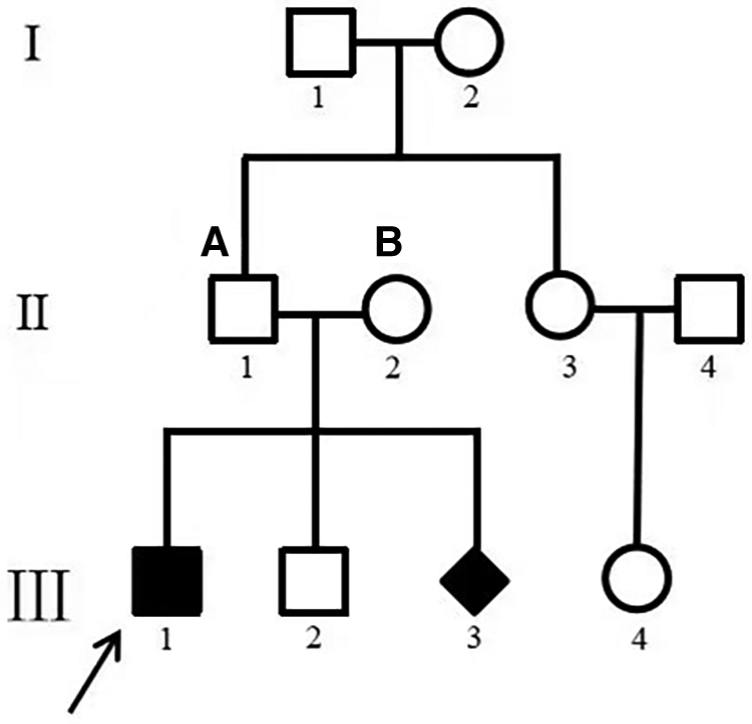
Family map. (**A**) GAA–G615V; (**B**) GAA–G802V. III 1 was patient 14. Pedigrees of the one family with phenotypic and genotypic information. I, II, and III refer to the first, second, and third generations of each family. Square symbols represent males; circle symbols denote females; Diamond symbol represents uncertain gender. Filled black symbols represent pompe patients; symbol with dots represent mutation carriers without clinical manifestation. White symbols denote unavailable family members. The arrows indicate the proband of each family. The genotype for each individual noted above the symbol where available. GAA, acid alpha-glucosidase.

Patient 14 was diagnosed as having myocardial hypertrophy, neonatal pneumonia and cardiac insufficiency with neonatal jaundice on the 8th day after birth. He died of heart failure at 3 months of age. All of the coding exons of the GAA gene were screened in this family using sequence-specific primers (Figure 6). A variant (c.2405G > T; p.G802V) in GAA was identified in the affected individual of the family (Table 3). Analyses with multiple bioinformatics tools predicted the GAA variant (p.G802V) to be deleterious. The residues are highly conserved among vertebrate species (Figure 3). His mother had GAA-G615V mutation, and father had GAA-G802V mutation respectively. Through his umbilical cord blood, the patient 14 was identified with compound heterozygous mutation of GAA-G615V/G802V (Figure 6). When this patient’s mother becoming pregnant again, we did prenatal genetic screening of the fetus. The fetus (III2) did not have any GAA mutations. We conducted a long-term regular follow-up of fetus III2 and found that the child was healthy and did not have hypertrophic cardiomyopathy. We also performed prenatal genetic testing on their third fetus. The fetus (III3) was identified as a heterozygous carrier of GAA-G615V/G802Vmutation (Figure 6) and died in the uterus.

## 4. Discussion

We described 15 patients with typical presentation of PD and fatal outcomes early in life. In this study, the cardiac characteristics of PD patients were comprehensively evaluated by analyzing the myocardial function data obtained from clinical, conventional echocardiography, serological biomarkers and GAA gene analysis. All patients showed a typical clinical course of infantile onset, characterized by hypertrophic cardiomyopathy and severe systemic weakness in the first few months of life, usually rapid progression and death at 1 year old.

Due to the low prevalence of Pompe disease, there is a scarcity of data focused on clinical characteristics, phenotype, and genetic characterization in China. Early recognition and diagnosis of Pompe disease is important. The clinical spectrum of PD depends on the age of onset. The severity of clinical manifestations, tissue impairment and age of onset correlate with the nature of mutations and the residual enzymatic activity levels (20). Classic infantile-onset Pompe disease (IOPD) is the most aggressive and life-threatening form of the disease.In classical IOPD, early manifestations of muscle weakness are often seen, as well as cardiomyopathy or cardiac hypertrophy (21). In contrast, late onset Pompe disease (LOPD) is generally associated with a wider range of age of onset and clinical symptoms. In our study, 80% (12 of 15 cases) patients presented with a typical clinical course of the infantile-onset form, which was characterized by hypertrophic cardiomyopathy and profound generalized weakness presenting in the first few months of life, with rapid progression and death usually occurring by one year of age. As IOPD patients continued to grow, they developed previously unrecognized complications of Pompe disease, such as ptosis, hyper-nasal speech, osteopenia, sensorineural and/or conductive hearing loss and gastroesophageal reflux (22). In our study, two patients were of late-onset form. The age of late onset was between 2 and 3 years old. They were complicated with hypertrophic cardiomyopathy, progressive lower limb weakness, and patient 11 had intellectual and hearing impairment.

The natural course of infantile Pompe disease has also been reported in a large retrospective chart review of 168 cases, including patients with atypical and classic infantile-onset form (23). In the large review study, the median age at symptom onset was 4 months, diagnosis at 4.7 months, and death occurred at 8.7 months. The average delay of diagnosis is 5.84 months in IOPD newborns with cardiomyopathy and other symptoms developed during the first 12 months of life. In PD patients with the onset of symptoms during the first 12 months of life, the longest delay of 109 months, was reported. A similar delay was observed in PD patients with the symptom onset between 12 months and 12 years (18, 20). In our study, the median age at symptom onset was 5.07 months and diagnosis at 19.53 months. All patients died during follow-up, and the median age at death was 24.80 months. The age at diagnosis was considerably older than that in the previous studies. Lack of awareness and qualified diagnostic tests might be the reason for the late diagnosis in Northwest of China. In this study, we found that the delay between symptom onset and diagnosis for Chinese patients with PD was similar to that in patients from the rest of the world. We feel that even with the use of blood-based assays there is still room for improvement in the delay of symptom onset and diagnosis. With the availability of specific tests, physicians can now diagnose Pompeii disease without invasive procedures. Improving understanding of the early symptoms and applying diagnostic tests of Pompe disease can prevent unnecessary invasive examinations such as muscle biopsy and achieve early diagnosis.

In our cohort, 6.7% (1 of 15 cases) of the patients was found having sensorineural hearing loss and mental retardation. Currently, hearing loss is recognized as an important cause of morbidity in IOPD patients. The auditory dysfunction of IOPD patients has been described in several studies; the most common type of hearing impairment is the sensorineural type (24–26). Chien Y-H et al. found that hearing loss and cognitive developmental delays were present in long-term survivors with IOPD (26). Van Capelle et al. reported on 11 IOPD patients who received Enzyme replacement therapy (ERT), 90.9% of them had hearing impairment (25). This may be explained by the less severe mutations and higher levels of residual *α*-glucosidase activity in juvenile patients (25). Martin et al. identified the central and peripheral nervous system dysfunction caused by glycogen storage for the first time through anatomopathological study (27). Hearing deficits were present shortly after birth in patients with IOPD. The extent of hearing loss did not decrease over time. This suggests that storage of glycogen in the cochlea had already commenced during pregnancy, and that enzyme therapy has no effect on it (25). It was also reported that weakness of the tensor veli palatini muscle might have caused a pressure drop in the middle ear, and may very well be the cause of the high incidence of conductive hearing loss found in infantile patients with Pompe disease (25, 28).

GAA sequencing is used to confirm PD diagnosis and identify the pathogenic variants. More than 900 different mutations have been identified in the GAA gene to date. Investigations have shown that the clinical course of Pompe disease is mainly established by the nature of the mutations in both GAA alleles, leading to different degrees of enzyme deficiency. In PD, as well as other genetic disorders, it is not easy to find a close correlation between genotype and phenotype. Up to 20% of mutations reported in GAA variant database are described without a strict correlation genotype/phenotype. PD patients with severe infantile form carry mutations that alter all forms of GAA causing low expression and enzymatic activity (18, 29, 30). The same mutations can be found in both infantile and late onset patients often with different incidence. GAA mutation analysis is used to confirm the diagnosis, assess the genotype−phenotype correlation, identify carriers within families, and provide genetic counseling (especially in IOPD families). In our study, we made a clear diagnosis of three children in a family, one of whom was diagnosed by postmortem umbilical cord blood (Patient 14), one was normal by amniocentesis, and one was identified as a heterozygous carrier of GAA-G615V/G802V mutation. In this paper, we characterized four novel mutations: c.2102T > C (p.L701P), c.2006C > T (p.P669l), c.766T > A (p.Y256N), and c.2405G > T (p.G802V). These affected amino acids were conserved in vertebrate genomes, making these mutations likely to be directly related to disease status. Nevertheless, the impact of new mutations should be assessed by functional studies.

According to previous studies, the frequency of gene mutations varies from country to country. PD is considered to be a pan-racial disease. However, the prevalence of the disease is very low in some countries. For example, in Finland, only one Pompe patient has been diagnosed with PD (31, 32). The c.-32-13T > G variant was the most common pathogenic mutation reported in California. The c.-32-13T > G splice mutation is very common in Caucasian patients with the childhood/adult form of the disease, with an allelic frequency ranging from 40% to 70% in different populations (33–36). Pompe disease-positive babies of Asian and Pacific Islander (API) heritage have a significant prevalence of the c.[1726G > A;2065G > A] variation (8, 37). The recent study found that there may be a potential high birth rate of Pompe disease in African Americans, but more data are needed to confirm (36). The c.525 del T and c.2481 + 102 _ 2,646 + 31 del mutations are highly prevalent in the Dutch population (35% and 31%, respectively) (38, 39). In contrast, the allele frequency of c.525 del T was 13.8% in Italian infants. In China, the most common pathogenic mutation in infants in northern China is c.2G > T (p. Glu888 *) mutation, accounting for 23.1% (40) of the total mutant alleles. The most common mutation in patients from southern China was c.1C > A (p.Asp 645Glu), accounting for 20%–25% of the total mutant alleles (20).

## 5. Limitation

Our study is a retrospective, single-center cohort with a relatively small sample size. In future studies, a large number of patients with Pompe disease will be important to verify the results of our survival analyses. The cut-off values determined in the current investigation may not be appropriate when utilizing a system different from our study since the parameter GLS is not yet standardized. In addition, this was a preliminary study and the number of cases included in the study was limited. More conclusions need to be answered by further multicenter prospective studies.

## 6. Conclusion

This study is one of the largest single-center studies regarding phenotypes as well as genotypes in Pompe disease in China. Infantile Onset Pompe Disease course was dominated by hypertrophic cardiomyopathy in patients with classic infantile disease. Its clinical spectrum strongly correlated with residual acid alpha-glucosidase activity. Residual activity of this enzyme is primarily determined by the severity of the pathogenic mutations on both GAA alleles and likely controlled by yet unknown modifying factors. Our study resulted in the discovery of 4 novel mutations. We emphasize the apparent benefits of early diagnosis. Finally, our study illustrated natural courses of PD patients in our cohort who were not treated by enzyme replacement therapy due to resource limitation at the time of our study.

## Data Availability

The datasets presented in this study can be found in online repositories. The names of the repository/repositories and accession number(s) can be found in the article/**Supplementary Material**.
