# Supplementary material for: Pompe disease in China: clinical and molecular characteristics
Source: Front Cardiovasc Med. 2023 Dec 14;10:1261172. doi: 10.3389/fcvm.2023.1261172 (PMC10755933; doi:10.3389/fcvm.2023.1261172)
Supplement: Supplementary file 1 [file Table1.docx]

Supplement table 1 Baseline demographics for the 15 patients with PD

| ID | Gender | Age at symptom onset (months) | Age of genetic diagnosis (months) | Age at death  (months) | Family history | Symptoms |
| --- | --- | --- | --- | --- | --- | --- |
| 1 | F | 4 | 10 | 11 | N | Muscle weakness, cardiomegaly |
| 2 | M | 3 | 4 | 6 | N | Shortness of breath, muscle weakness, cardiomegaly |
| 3 | M | 2 | 3 | 4 | N | Muscle weakness, cardiomegaly, heart failure |
| 4 | M | 4 | 5 | 60 | Y | Motor retardation, cardiomegaly |
| 5 | M | 2 | 3 | 10 | Y | Recurrent airway infections, feeding difficulties, cardiomegaly, heart failure |
| 6 | F | 2 | 3 | 5 | Y | Feeding difficulties, drowsiness, shortness of breath, muscle weakness, cardiomegaly |
| 7 | F | 9 | 10 | 11 | N | Shortness of breath, glossomegaly |
| 8 | M | 1 | 3 | 3 | N | Recurrent airway infections, cardiomegaly, heart failure |
| 9 | F | 2 | 4 | 7 | Y | Respiratory distress, cardiomegaly, congestive heart failure |
| 10 | M | 1 | 3 | 6 | Y | Pneumonia, fever, weight loss |
| 11 | F | 12 | 108 | 120 | N | Lameness, strephenopodia, language disorder, moderate mental retardation, poor hearing, short stature |
| 12 | F | 5 | 5 | 7 | N | Recurrent airway infections, feeding difficulties, cardiomegaly |
| 13 | M | 4 | 5 | 6 | N | Muscle weakness, recurrent airway infections, cardiomegaly |
| 14 | M | 1 | 15 months after death, umbilical cord blood | 3 | N | Neonatal jaundice, respiratory tract infection, heart failure |
| 15 | F | 24 | 109 | 113 | Y | Muscle weakness, recurrent airway infections, cardiomegaly |

PD, Pompe disease; ID, identity; F, female; M, male; N, no; Y, yes.

Supplement table 2 Biochemical indicators of the 15 patients with PD

| ID | ALT  (IU/L) | AST  (IU/L) | CK  (IU/L) | CK-MB  (IU/L) | LDH  (IU/L) | NT-proBNP  (pg/ml) | GAA |
| --- | --- | --- | --- | --- | --- | --- | --- |
| 1 | - | - | - | - | - | - | - |
| 2 | - | - | - | - | - | - | - |
| 3 | - | - | - | - | - | - | - |
| 4 | 158 | 199 | 450 | - | - | 321 | - |
| 5 | 49 | 76 | 1888 | 51.48 | 386 | 8231 | - |
| 6 | 131 | 175 | 552 | - | - | 7002 | 9.1nmol/g/min (24.8-93.0) |
| 7 | 155 | 227 | 483 | 21.5 | 1183 | 7600 | 3.4nmol/g/min  (24.8～93.0) |
| 8 | 99 | 151 | 469 | - | - | 10611 | - |
| 9 | - | - | - | - | - | - | - |
| 10 | - | - | - | - | - | - | - |
| 11 | 117 | 225 | 1085 | 18.9 | 836 | 275 | 1.5nmol/L/min  (≥6.5) |
| 12 | 159 | 314 | 1031 | 15.1 | 1338 | 3665 | - |
| 13 | 150 | 268 | 815 | 34 | 202 | 13654 | 0.1μmol/L/h  (1.46-20.34) |
| 14 | 100 | 153 | 453 | - | - | 30802 | - |
| 15 | 155 | 212 | 1632 | 40.4 | 779 | 135 | 0.18μmol/L/h  (1.46-20.34) |

PD, Pompe disease; ID, identity; ALT, alanine aminotransferase; AST, aspartate aminotransferase; CK, creatine kinase; CK-MB, MB isoenzyme of creatine kinase; LDH, lactate dehydrogenase; NT-proBNP, N-Terminal Pro-Brain Natriuretic Peptide

Supplement figure 1 Cutoff Value for GLS. 7.6% cutoff value for GLS determined by X-Tile software.


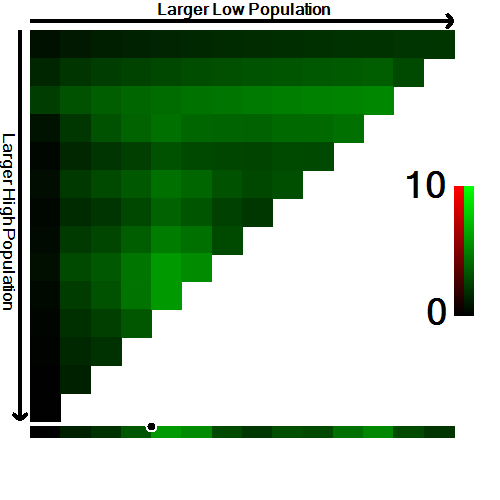


GLS, global longitudinal strain
