# Supplementary figures and images for: Pompe disease in China: clinical and molecular characteristics
Source: Front Cardiovasc Med. 2023 Dec 14;10:1261172. doi: 10.3389/fcvm.2023.1261172 (PMC10755933; doi:10.3389/fcvm.2023.1261172)

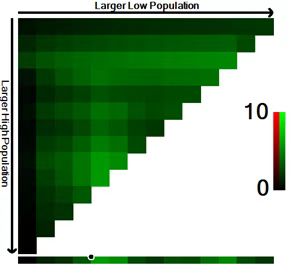

Supplement: Supplementary file 2 [file Image1.jpeg]
